# Delta-catenin attenuates medulloblastoma cell invasion by targeting EMT pathway

**DOI:** 10.3389/fgene.2022.867872

**Published:** 2022-10-11

**Authors:** Yuanjun Hu, Sihan Zhu, Rizhen Xu, Manxia Wang, Furong Chen, Zeshun Zhang, Binghong Feng, Jian Wang, Zhongping Chen, Jing Wang

**Affiliations:** ^1^ State Key Laboratory of Oncology in South China, Collaborative Innovation Center for Cancer Medicine, Guangzhou, China; ^2^ Department of Neurosurgery/Neuro-Oncology, Sun Yat-sen University Cancer Center, Guangzhou, China; ^3^ Department of Neurosurgery, The Third Affiliated Hospital of Sun Yat-sen University, Guangzhou, China; ^4^ Department of Surgery, Guangzhou University of Chinese Medicine, Guangzhou, China; ^5^ Department of Pharmacology, Guangdong Pharmaceutical University, Guangzhou, China

**Keywords:** medulloblastoma, prognosis, invasion, delta-catenin, EMT

## Abstract

**Background:** Medulloblastoma is the most common pediatric malignant tumor in central nervous system. Although its prognosis has been improved enormously by the combination treatments with surgery, radiotherapy, and chemotherapy, it still could progress *via* invasion and distant dissemination. We aimed to investigate molecular mechanisms of medulloblastoma invasion in the current work.

**Methods:** The gene expression profile of medulloblastoma were analyzed based on the data deposited in Gene Expression Omnibus (GEO) and filtered according to brain specific proteins in the Uniprot. Delta-catenin was identified and further analyzed about its expression and roles in the prognosis of medulloblastoma patient. The function of delta-catenin on cell invasion and migration were investigated by transwell and wound healing assay. Whether delta-catenin participates in the epithelial-mesenchymal transition (EMT) regulated invasion was also studied.

**Results:** Delta-catenin expression was highly upregulated in tumor tissues compared to normal tissues from medulloblastoma patients in five independent, nonoverlapping cohorts. Furthermore, delta-catenin expression level was upregulated in WNT subgroup, and significantly correlated with better prognosis, and associated with metastasis through GEO database analysis. Functional assays indicated that delta-catenin inhibited medulloblastoma cell invasion and migration through regulating the key factors of EMT pathway, such as E-cadherin and vimentin.

**Conclusion:** Delta-catenin might be a positive predictor for prognosis of medulloblastoma patients, through attenuating medulloblastoma cell invasion by inhibiting EMT pathway.

## Introduction

Medulloblastoma is the most common malignant pediatric brain tumor. In recent decades, therapeutic strategy and prognosis have been based on risk stratification of medulloblastoma patients by age at presentation, extent of tumor resection, and tumor metastases. Molecular profiling has revealed four molecular subgroups of medulloblastoma—wingless (WNT), sonic hedgehog (SHH), Group 3, and Group 4—with distinct molecular and clinical profiles (Northcott et al., 2011; Northcott et al., 2012; Coltin, 2021). The latest WHO classification of tumors of the central nervous system simplified medulloblastoma into three subgroups—WNT, SHH and non-WNT/non-SHH (Louis et al., 2021). Although this has improved the guidance for treatment, medulloblastoma invasion and dissemination occurs in all subgroups, and is still the major cause of medulloblastoma deaths (Fults et al., 2019). Leptomeningeal dissemination of medulloblastoma has long been considered to occur *via* cerebrospinal fluid, and hematogenous transfer has recently been described (Garzia et al., 2018). Metastases are found in 5%–45% of medulloblastoma cases at initial diagnosis, least often in WNT subgroup (5–10%) and most often in Group 3 (40–45%) (Juraschka and Taylor, 2019). Although nearly all medulloblastoma cases ultimately progress to metastasis *via* invasion and dissemination, this clinical challenge remains the least understood aspect of medulloblastoma pathogenesis and disease progression.

Delta-catenin, a catenin encoded by CTNND2, is a known neuroprotein that interacts with presenilin-1 (Zhou et al., 1997). Actually, abnormal function of delta-catenin is associated with several neurodevelopmental disorders, such as cri-du-chat syndrome, autism and schizophrenia (Medina et al., 2000; Vrijenhoek et al., 2008; Turner et al., 2015). The important role of delta-catenin in neurodevelopment may related with its function on dendrite development (Martinez et al., 2003; Arikkath et al., 2008). Recent work has revealed delta-catenin is overexpressed in a series of peripheral tissue neoplasms such as prostate and lung, suggesting its value as a cancer biomarker (Lu et al., 2014). Delta-catenin was mainly studied as a oncoprotein in several tumor types (Huang et al., 2018; Shen et al., 2021; Zeng et al., 2009; Zhang et al., 2015). However, the anti-tumor property of delta-catenin was also reported in a few studies (Westbrook et al., 2005; Frattini et al., 2013). Delta-catenin is a member of the p120-catenin family of catenin protein and it could bind to E-cadherin in competition with p120ctn, which belongs to the same family, indicating that it may participate in cell adhesion and EMT (Reynolds and Roczniak-Ferguson, 2004; Zhang et al., 2014). EMT, which involves changes in morphology and increased cell motility, is currently considered to be a major pathway in metastasis. Because medulloblastoma is a neuroepithelial-derived tumor, its invasion and dissemination are likely inseparable from EMT (Fults et al., 2019).

Although the role of delta-catenin has been examined in glioma (Shimizu et al., 2019; Wang et al., 2011), there are no prior reports of its role in medulloblastoma. We therefore aimed to elucidate the molecular mechanisms of medulloblastoma invasion, to improve the therapeutic response and prolong survival in medulloblastoma patients. We examined delta-catenin expression in medulloblastoma, and its effect on invasion and dissemination, using bioinformatic analysis and *in vitro* experiments. Delta-catenin expression was associated with improved survival. Delta-catenin alleviated medulloblastoma invasion *in vitro* by targeting EMT pathway.

## Materials and methods

### Bioinformatics analysis

The Differentially Expressed Genes (DEGs) were analyzed using the GEO database (GSE74195, GSE66354, and GSE86574) by “limma” R package. After Benjamini–Hochberg (BH) multiple test adjustment, DEGs with absolute log_2_ fold change (FC) > 1 and *p* < 0.05 were considered to be included for subsequent analysis. Then the DEGs were considered as delta-catenin related compared with the brain specific protein list in the Uniprot.

We further compared delta-catenin expression use GEO datasets between normal and medulloblastoma patient tissues. Then we used the clinical information from GEO datasets and the medulloblastoma patient cohort (74) from Sun Yat-sen University Cancer Center (SYSUCC, Guangzhou, China) (Table 1) to analyze the relevance between delta-catenin expression and clinical characteristics such as prognosis, molecular subgroup and metastasis. The patients data from GSE85217 were divided into two independent nonoverlapping cohorts (7:3 ratio) for mutual verification (Chen et al., 2021; Li et al., 2020). All the GEO datasets used in the bioinformatics analysis were listed in Supplementary Table S1.

**TABLE 1:** Summary of clinical information of medulloblastoma cohorts.

	GSE85217 dataset 1	GSE85217 dataset 2	GSE30074	GSE21140	SYSUCC cohort
Case included (*n*)	534	229	30	103	74
Age (mean ± SD)	5.6 (±8.01)	8.6 (±7.39)	NA	9.4 (±8.39)	11.3 (±8.62)
Overall survival	N = 429	N = 183	N = 30	NA	N = 74
Year (mean ± SD)	4.77 (±3.70)	5.41 (±3.92)	4.25 (±2.29)	—	3.52 (±3.55)
Status (alive/dead)	309/120	144/39	18/12	—	44/30
Gender	N = 411	N = 183	N = 30	N = 103	N = 74
Male	288	132	19	63	52
Female	123	51	11	40	22
Subgroup	N = 763	NA	NA	N = 103	NA
WNT	70	—	—	8	—
SHH	223	—	—	33	—
Non-WNT/SHH	470	—	—	62	—
Metastasis	N = 400	N = 172	NA	NA	NA
M0	273	124	—	—	—
M1	127	48	—	—	—

Abbreviation: SHH, sonic hedgehog; WNT, wingless; M0, Non-Metastasis; M1, metastasis; SYSUCC, Sun Yat-sen University Cancer Center.

The bioinformatics analysis was conducted mainly in R v. 38.0. The “combat” function of the “sav” R package was used for batch-effect correction. The “Coxph” function, and the “survival” R package, were used to estimate the relationship between delta-catenin expression and survival. We used R package “survivalROC” to plot the receiver operating characteristic (ROC) curve of overall survival and calculate the area under the curve (AUC). 0.5 generally indicates some predictive ability. The higher the AUC is, the more accurate the prediction result is. BioGRID7 (https://thebiogrid.org/) is a biomedical interaction repository. The database can be used to retrieve publications on protein and genetic interactions, chemical interactions and post-translational modifications of important model organism species. Gene set enrichment analysis (GSEA) was performed using the GSEA software and its results were visualized using “Cluster Profiler” and “ggplot2” in R.

### Cell culture and patient specimens

The medulloblastoma cell lines Daoy and ONS-76, and a tool cell line HEK293T were obtained from the Cell Bank of State Key Laboratory of Oncology in South China. The cells were cultured in DMEM with 10% fetal bovine serum (Gibco, Waltham, MA, United States) in a humidified incubator at 37°C with 5% CO_2_. All the specimen from patients treated in our institute were residuals after surgery. The patients were all informed and provided signed consent regarding the use of their biological specimens and clinical information for research purposes. This study was approved by the ethics committee and institutional review board of Sun Yat-sen University Cancer Center (SYSUCC, Guangzhou, China), in accordance with the Helsinki Declaration.

### Immunohistochemistry and scoring

Paraffin-embedded tissue samples (*n* = 74) from medulloblastoma patients who underwent surgery at Sun Yat-sen University Cancer Center from 2011 to 2020 were used for immunohistochemical staining (IHC). Medulloblastoma diagnosis was based on the World Health Organization Classification of Central Nervous System Tumors (2016). Delta-catenin expression were detected by immunohistochemistry following standard protocol as per our previous paper (Wang et al., 2022). Briefly, the paraffin-embedded tissue slides were first heated at 65°C for 2 h, then sequentially deparaffinized in xylene, rehydrated *via* an ethanol gradient, antigen-retrieved using citric acid buffer (pH 6.0), and blocked with goat serum. Tissue samples were incubated overnight at 4°C with the antibody against delta-catenin (Cat# sc-81793; Santa Cruz Biotechnology, Inc.). HRP-labeled goat anti-rabbit/mouse antibody was then added to the slides for 1 h at 26°C. Finally, the tissues were stained with diaminobenzidine (DAB) and counterstained with hematoxylin. The slides were visualized and photographed using an automatic slide scanner (KF-PRO-020) at ×40 magnification. Quantitative image analyses were conducted using the HALO software (Indica labs, Corrales, MN, United States) using the multiplex IHC modules (Sun et al., 2019). The histochemistry score was used as the grouping criterion.

### Lentiviral infection

To establish stable knockdown and overexpression (OE) lines of Daoy and ONS-76, the plasmids pSlenti-U6-shRNA (CTNND2)-CMV-EGFP-F2A-Puro-WPRE (OBiO Technology, Shanghai, China) and pEZ-Lv105-hCTNND2-Puro (GeneCopoeia, Guangzhou, China) were used. The plasmid was co-transfected with lentivirus packaging plasmids (PLP1, PLP2, and VSVG; Invitrogen, United States) into HEK293T with transfection reagent Lipofectamine 3000 (L3000008, Thermo Fisher Scientific, United States). The supernatant of HEK293T was collected twice, at 48 and 72 h after transfection. Cells (Daoy and ONS-76) were infected with the filtered supernatant containing virus. Puromycin (2 μg/ml) was added for 48 h after the infection and selected for 7 days. The target sequence for delta-catenin was 5′-GCA​ACA​ACA​CTG​CAA​GCA​A-3′ and 5′-GCT​AAA​GGC​GAA​CAC​ACT​T-3′.

### Real-time PCR

Total RNA from the cell lines was extracted following the protocol of RNA-Quick Purification Kit (ES Science, China). The reverse transcription were performed with HisScript III All-in-one RT SuperMix (Vazyme Biotech Co., Ltd.). Then real-time PCR was performed using ChamQ SYBR qPCR Master Mix (Vazyme Biotech Co., Ltd. China), and triplicate samples were run on a Bio-Rad CFX96 qPCR system according to the manufacturer’s protocol. The Ct values for delta-catenin was normalized to β-actin, and the 2^−ΔΔCt^ method was used for quantitative analysis. The primer sequences for delta-catenin were: 5′-AGG​TCC​CCG​TCC​ATT​GAT​AG-3′ and 5′-ACT​GGT​GCT​GCA​ACA​TCT​GAA-3′. The sequences for β-actin were: 5′- CTC​CAT​CCT​GGC​CTC​GCT​GT-3′ and 5′- GCT​GTC​ACC​TTC​ACC​GTT​CC-3′.

### Western blotting

Cells were collected and washed twice in PBS, lysed in RIPA buffer (Biotime Biotechnology, Shanghai, China) with PMSF on ice for 30 min, then centrifuged at 12,000 × g for 15 min at 4°C. Protein concentration was determined with the BCA kit (Cat# 23227; Thermo Fisher Scientific). And the lysates were then subjected to SDS-PAGE (10% gel). The separated protein bands were transferred onto PVDF membrane (IPVH00010, Millipore, United States). After been blocking in milk (5% in TBST) for 1 h at 26°C, the membrane was probed with primary antibodies, such as for delta-catenin (ab184917; Abcam), E-cadherin (Cat# 3195; cell signaling technology), and vimentin (Cat# 10366-1-AP; Proteintech), beta-tubulin (Cat# 2128S, CST) and GAPDH (Cat# 2118S, CST) at 4°C for 12–16 h. After been washed in TBST for three times (15 min each time), the membranes were incubated with corresponding HRP-conjugated secondary antibodies (Cat# 7074S and Cat# 7076S, CST) for 1 h at 26°C. The bands were visualized using Ncm-ECL Ultra (New Cell & Molecular Biotech Co., Ltd., China), captured using an ChemiDoc Touch Imaging System (Bio-Rad, United States), and quantified using ImageJ (v1.8.0).

### Immunofluorescence staining

Daoy (shNC and sh delta-catenin) cells were seeded in confocal dishs 24 h in advance. Then the dishs were fixed with 4% formaldehyde for 15 min at 26°C, rinsed three times in PBS (5 min each time), and blocked in blocking buffer (PBS with 5% v/v normal goat serum, 0.3% v/v Triton X-100) at 26°C for 60 min. The cells were then incubated overnight at 4°C in diluted primary E-cadherin antibody (1:100, Cat# 3195; CST). Then the slides were rinsed three times in PBS for 5 min each and incubated with fluorochrome-conjugated secondary antibody (diluted in blocking buffer at 1:500, Cat# 8889S, CST) for 1 h at 26°C in dark. The slides were rinsed three times again in 1× PBS for 5 min each, and DAPI was added to counter-stain the nucleus. Images were obtained using a Zeiss LSM 880 with fast Airyscan.

### Cell migration and invasion assay

Cells were suspended in serum-free DMEM (2.5 × 10^5^ cells/ml), 200 µl was inoculated into the upper chamber and 700 µl of complete medium was simultaneously added to the lower chamber. When placing the chamber, avoid air bubbles attached to the bottom of the chamber. After culturing for 15 h in incubator, the upper chamber was fixed in 4% paraformaldehyde for 30 min, then stained with 0.5% crystal violet for 7 min. The images were captured by the microscope (Nikon ECLIPSE Ni) when the chambers were dried and then counted the number of cells by ImageJ software (v1.8.0). For the invasion assay, 0.2% matrigel (Cat# 356234, Corning, NY, United States) was added into upper chamber before cells were seeded. For delta-catenin-overexpression (OE) cells, more cells (1 × 10^6^ cells/ml) were suspended and culturing time extended to 24 h.

### Wound healing assay

Cells were inoculated in 6-well plate (5 × 10^5^ cells/well) and then the plates were placed in the incubator for pre-culture (at 37°C, 5% CO_2_) overnight. Scratching on the cell surface with tip vertically next day and washing three times with PBS, then adding serum-free medium and placing in the incubator. Capturing images at the same point for each group in 0, 12, 24, and 48 h. The healing area between scratches were measured by ImageJ software (v1.8.0).

### Statistical analysis

All the cell culture experiments were performed three independent replicates. Statistical analyses were performed using SPSS version 23.0 (IBM Corp, Armonk, NY) and GraphPad Prism version 8.0 (La Jolla, CA, United States). Survival analyses were performed using the Kaplan-Meier survival curves. All the data are shown as mean and standard deviation (mean ± SD). *p* < 0.05 was considered statistically significant for Student’s t-tests or one-way ANOVA.

## Results

### Delta-catenin is identified by a filtering strategy

**FIGURE 1:**
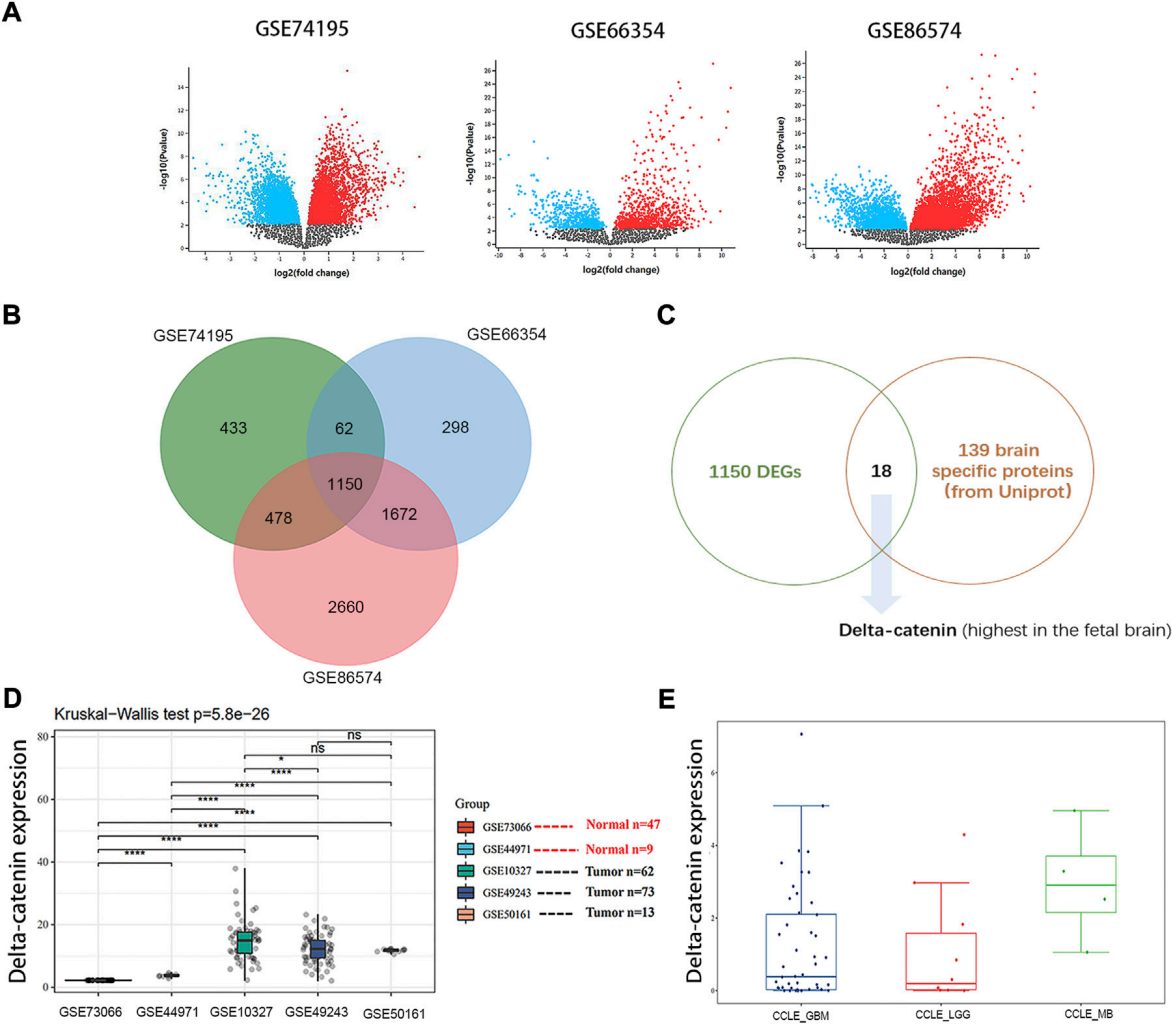
Delta-catenin is identified by a filtering strategy. **(A)** DEGs for medulloblastoma in three independent, nonoverlapping datasets were identified. **(B)** The overlap of the three datasets contains 1150 DEGs. **(C)** Flow diagram for screening out delta-catenin. **(D)** Delta-catenin expression is particularly high in medulloblastoma tissue compared to normal tissue controls (*p* < 0.0001) in five independent nonoverlapping cohorts. **(E)** Delta-catenin expression in various brain tumor cell lines (e.g., U87 and U251 for GBM, SF767 and SW1088 for LGG, Daoy and D283 for MB).

Several thousand differentially expressed genes were pulled out in the following datasets, 2123 in GSE74195, 3182 in GSE66354 and 5960 in GSE86574 (Figure 1A). The overlap of the three datasets contains 1150 DEGs (Figure 1B). Given that the cell origin of medulloblastoma is cerebellum and different subtypes arise within the distinct cell origins (Gibson et al., 2010), those brain specific proteins were more relevant to the development of medulloblastoma. Eighteen candidates were selected based on 139 brain specific proteins from Uniprot (Supplementary Table S2) and 1150 DEGs (Supplementary Table S3). Considering that medulloblastoma mainly occurs in children and the expression of delta-catenin is high in fetal brain (Turner et al., 2015), we then determined to further study the role of delta-catenin (Figure 1C).

### Delta-catenin is verified highly expressed in medulloblastoma tissues and cells

Gene Expression Profiling Interactive Analysis (GEPIA) is an interactive web application for gene expression analysis based on 9736 tumors and 8587 normal samples from the TCGA and the GTEx databases using the output of a standard processing pipeline for RNA sequencing data (Tang et al., 2017; Tang et al., 2019). Using online GEPIA, we analyzed expression of delta-catenin between tumor and normal tissue in various cancer types (Supplementary Figure S1). Delta-catenin expression was significantly higher in normal brain tissues and glioma patient tissues (both low-grade glioma and glioblastoma), than in other tumor types, supporting that delta-catenin may be a neural-specific protein. However, there are no medulloblastoma data in the GEPIA database which is based on TCGA and GTEx datasets. We then turned to GEO database, and examined the clinical significance of delta-catenin in medulloblastoma, 56 normal and 211 medulloblastoma samples (non-paired) were included. Its expression was significantly upregulated in medulloblastoma compared to the normal controls (*p* < 0.0001). Delta-catenin expression in medulloblastoma cell was comparable with that in glioblastoma and low-grade glioma cells (Figures 1D,E).

### High delta-catenin expression is a favorable prognostic factor in medulloblastoma

**FIGURE 2:**
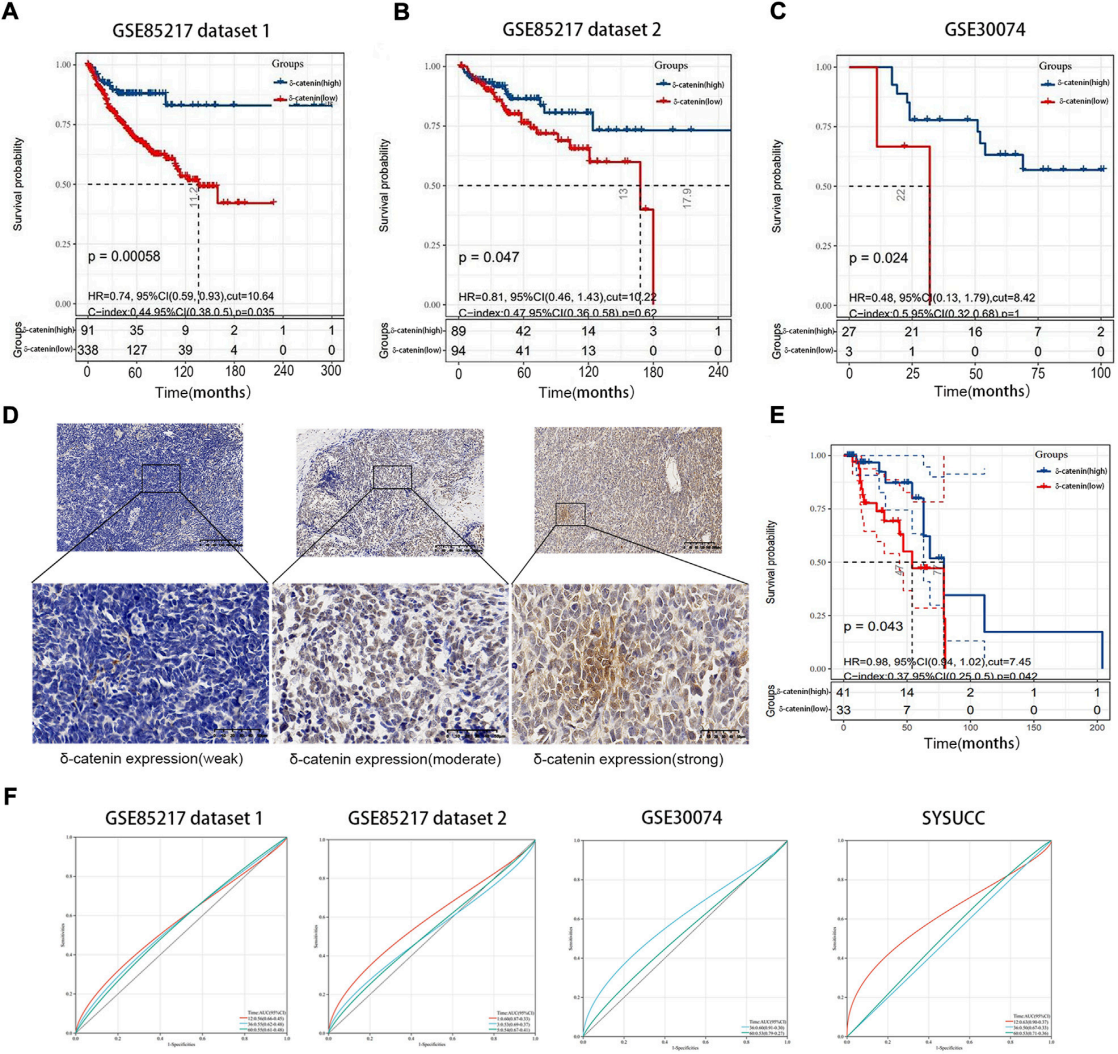
Delta-catenin expression and relevance for medulloblastoma prognosis. **(A-C)** Kaplan-Meier curves for medulloblastoma patients, based on delta-catenin expression. Data obtained from two nonoverlapping cohorts. **(D)** Immunohistochemistry reveals weak to strong delta-catenin expression in medulloblastoma tissues. **(E)** Kaplan-Meier curve for 74 medulloblastoma cases at the Sun Yat-sen University Cancer Center (SYSUCC), according to delta-catenin expression. **(F)** ROC curves of delta-catenin expression on the overall survival rate in four cohorts and AUC corresponding to each curve. The red, blue, and green lines represent the ROC curve of 1-year, 3-year, and 5-year overall survival rates.

Using data from two independent and nonoverlapping medulloblastoma patient cohorts, we examined the association between delta-catenin expression level and medulloblastoma prognosis. We divided the Cavalli cohort dataset (GSE85217) (Cavalli et al., 2017) into two groups (7:3 ratio) for analysis. Overall survival analysis of medulloblastoma patients revealed that higher delta-catenin expression was directly associated with favorable overall survival (Figures 2A–C). To further confirm the finding, we detected delta-catenin expression in 74 medulloblastoma patients recruited in our institute, the typical staining pattern were shown in Figure 2D. Similarly, high delta-catenin expression was associated with better prognosis (Figure 2E). These findings suggest that delta-catenin is a favorable predictor for medulloblastoma patients. ROC curve and AUC values reflect diagnostic values of markers. We drew 1-, 3-, and 5-year ROC curves of delta-catenin expression on the overall survival rate in four cohorts and calculated the AUC corresponding to each curve. The AUCs of the 1-, 3-, and 5-year GSE85217 dataset1 cohorts were 0.56, 0.55, and 0.55, respectively. The AUCs of the 1-, 3-, and 5-year GSE85217 dataset2 cohorts were 0.60, 0.53, and 0.54, respectively. The AUC values of the 3-, and 5- year GSE30074 cohorts were 0.60 and 0.53, respectively. The AUC values of the 1-, 3-, and 5-year SYSUCC cohorts were 0.63, 0.50, and 0.53 (Figure 2F).

### Delta-catenin is enriched in WNT subgroup and is associated with dissemination

**FIGURE 3:**
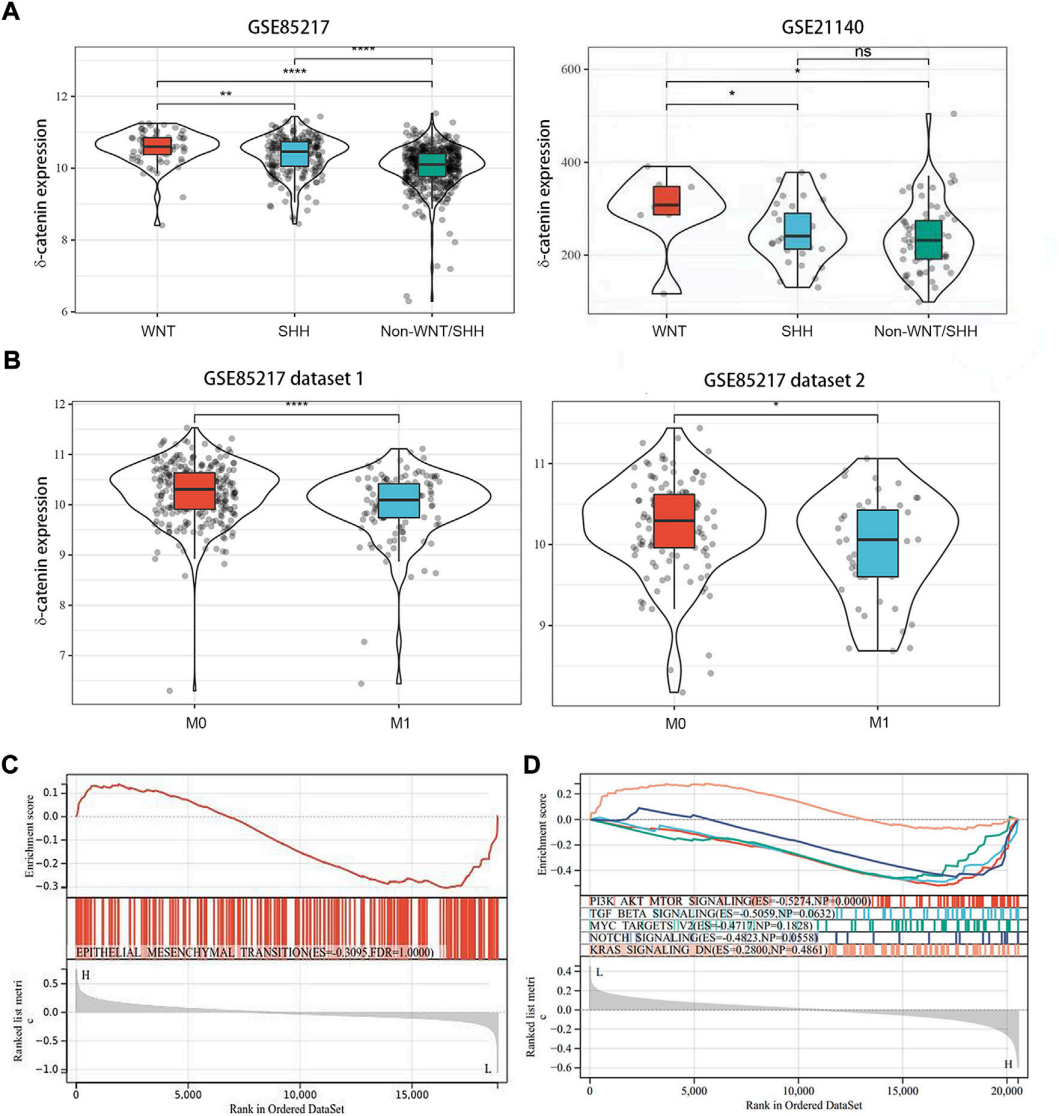
Delta-catenin expression in the medulloblastoma molecular subgroups, and its effects on metastasis. **(A)** Delta-catenin expression by medulloblastoma subgroup: delta-catenin was enriched in WNT subgroup. Data obtained from three nonoverlapping cohorts. SHH: sonic hedgehog; WNT: wingless. **(B)** Delta-catenin mRNA expression in tumor samples from non-metastatic and metastatic tumors. Delta-catenin expression is significantly higher in non-metastatic than in metastatic samples (Student’s *t*-test). **(C)** GSEA of genes whose expression is downregulated by delta-catenin reveals the over-representation of EMT-signaling-related genes. **(D)** GSEA analysis showed that the metastasis-related signaling pathways such as PI3K/AKT, TGF-beta and so on were highly enriched in the low delta-catenin expression group.

In our study, delta-catenin expression was significantly higher in WNT group of medulloblastoma than in non-WNT groups (SHH, Group 3, and Group 4) (Figure 3A) based on two datasets GSE85217 and GSE21140. Metastasis frequency was, in fact, lowest in WNT subgroup in medulloblastoma patients (Fults et al., 2019). Based on these two findings, we speculate that delta-catenin participates in regulating medulloblastoma invasion and dissemination. Interestingly, data of the cohorts from Cavalli (Cavalli et al., 2017), confirmed that high delta-catenin expression was associated with low tumor dissemination (Figure 3B). Based on GSEA analysis, the EMT molecules were highly enriched in the low-delta-catenin expression group (Figure 3C). In addition, we also enriched some signaling pathways that have been widely reported to affect the metastasis of medulloblastoma, such as PI3K/AKT, TGF-beta, MYC, Notch, KRAS signaling pathway (Li et al., 2021). The metastasis-related signaling pathways were also enriched in the low delta-catenin expression group (Figure 3D). These findings supports our hypothesis that delta-catenin suppresses both invasion and migration in medulloblastoma. In addition, the BioGRID database showed interactions between delta-catenin and a large number of proteins. The most significant correlations were EGFR, CLK1, ATN1, LNX1, TRIM47, NUDT12, TTR, ESR1, KAT28, MCM2, PTGER4, THUMPD3, PDZD2, LRRC7, ZMYND19, NR3C1 and ZBTB33 (Supplementary Figure S2).

### Delta-catenin attenuates medulloblastoma cell invasion and migration

**FIGURE 4:**
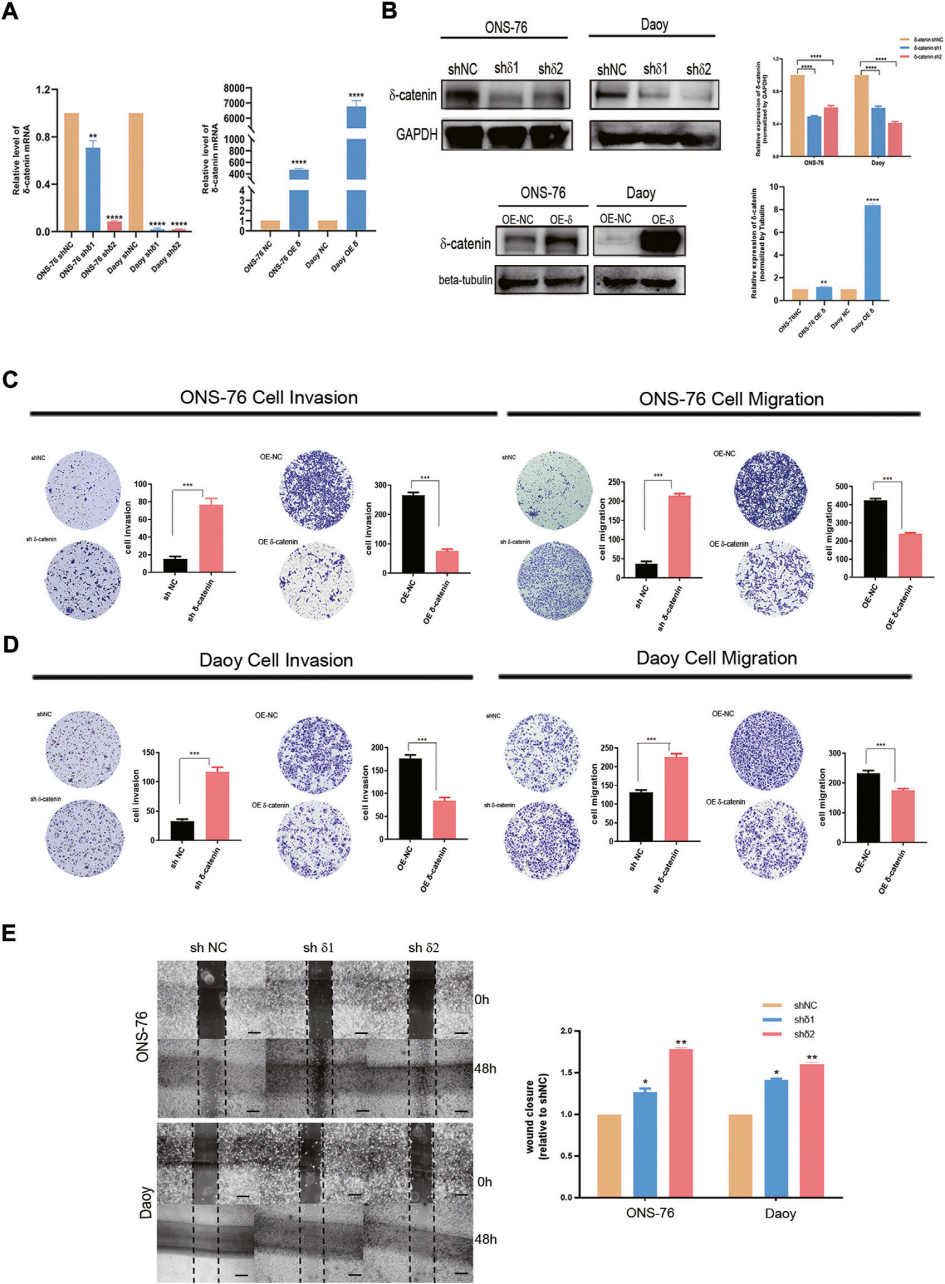
Effects of delta-catenin knockdown and overexpression in Daoy and ONS-76 cells migration and invasion. **(A)** Real-time quantitative PCR analysis of delta-catenin mRNA following lentiviral transfection. β-actin was used as a control. **(B)** Western blot analysis of delta-catenin expression following lentiviral transfection. GAPDH and Tubulin were used as controls. **(C)** Representative images (×10 magnification) of invasion and migration by delta-catenin-knockdown and overexpressing ONS-76 cells. **(D)** Representative images (×10 magnification) showing invasion and migration by delta-catenin-knockdown and overexpressing Daoy cells. **(E)** Cellular migration in both the sh#1 and sh#2 groups of ONS-76 and Daoy cells were promoted, as determined *via* a wound-healing assay. Scale bars, 100 μm.

To evaluate the role of delta-catenin expression in medulloblastoma dissemination, we established stable knockdown and OE cells of Daoy and ONS-76. RT-qPCR and western blotting were used to confirm infection efficiency (Figures 4A,B). Transwell assay was applied to test the effect of delta-catenin on medulloblastoma invasion and migration. In both medulloblastoma cell lines, delta-catenin-knockdown significantly promoted medulloblastoma invasion and migration, whereas delta-catenin overexpression attenuated them (Figures 4C,D). Wound healing assay also showed that delta-catenin-knockdown accelerated medulloblastoma migration (Figure 4E). Actually, we also found that delta-catenin could inhibit the proliferation of medulloblastoma cells (Supplementary Figure S3). These are consistent with our bioinformatics findings, and supports that delta-catenin affects medulloblastoma invasion and dissemination *in vitro*.

### Delta-catenin attenuates medulloblastoma cell invasion and migration by targeting epithelial-mesenchymal transition

**FIGURE 5:**
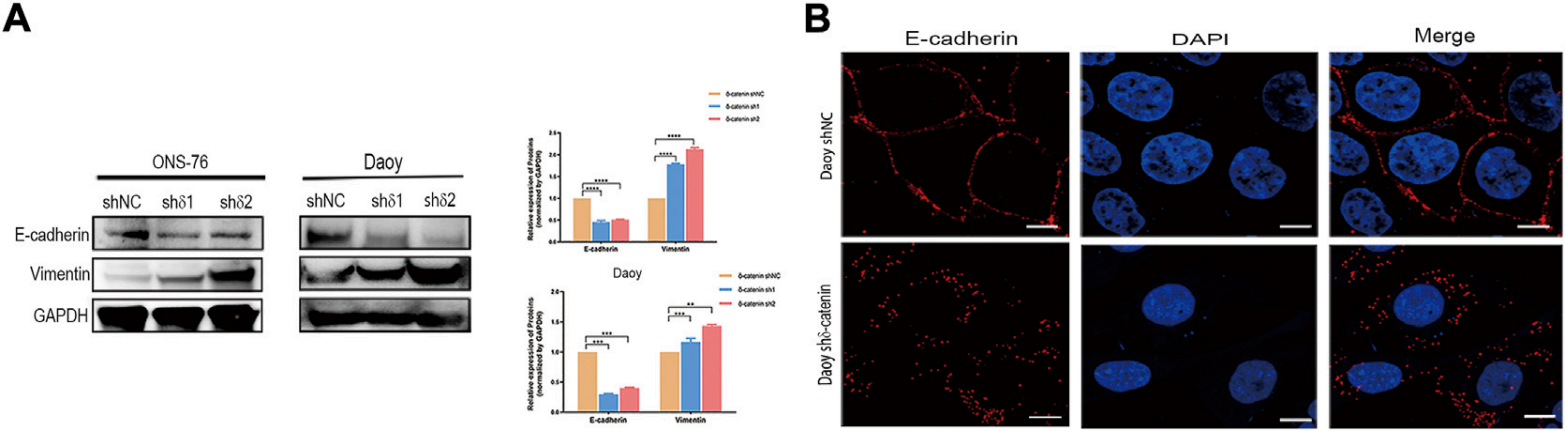
Delta-catenin attenuates medulloblastoma cell migration and invasion by targeting EMT pathway. **(A)** Western blot analysis of EMT pathway markers (E-cadherin and vimentin) following lentiviral transfection of ONS-76 and Daoy cells. GAPDH was used as a control. **(B)** Representative images (×100 magnification) of E-cadherin in Daoy sh delta-catenin and control cell lines, *via* immunofluorescence staining. Scale bars, 5 μm.

Next, we examined how delta-catenin inhibits medulloblastoma invasion and migration, and hence metastasis, by observing how it affects EMT pathway, a major pathway of metastasis. We detected the expression of EMT markers (E-cadherin and vimentin) in delta-catenin-knockdown medulloblastoma cells and control cells. Delta-catenin knockdown significantly reduced the expression of E-cadherin and increased that of vimentin (Figure 5A). Moreover, *Via* immunofluorescence, we found that knockdown of delta-catenin broke down the continuous arrangement of E-cadherin in the adjacent areas of cell to discrete and disorganized form (Figure 5B). These data indicated that delta-catenin regulated both the quantity and structure of E-cadherin, and therefore attenuate medulloblastoma cell invasion and migration *via* EMT pathway.

## Discussion

We aimed to elucidate the molecular mechanisms of medulloblastoma invasion, to improve the therapeutic response and prolong survival in medulloblastoma patients. Our bioinformatics analysis revealed that delta-catenin expression was positively associated with survival of medulloblastoma patients. It was enriched in WNT subgroup, which had the lowest metastasis rate among the four subgroups. Delta-catenin inhibited medulloblastoma cell invasion *in vitro*, and EMT pathway may be the underlying mechanism.

These findings are consistent with various studies showing that delta-catenin inhibits tumors. For example, CTNND2 loss-of-function mutation was found to be common in glioblastoma. Based on analysis of TCGA data, reduced CTNND2 expression was associated with poor prognosis of glioblastoma, especially for the mesenchymal type (Frattini et al., 2013). Delta-catenin inhibited glioma cell proliferation and self-renewal, followed by phenotypic transformation from the aggressive mesenchymal cell type to the neuronal cell type (Frattini et al., 2013). Among 25 identified genes that potentially suppress EMT, CTNND2 was the most likely to suppress EMT (Westbrook et al., 2005).

However, delta-catenin was also reported to serve as an oncoprotein (Huang et al., 2018; Shen et al., 2021; Zeng et al., 2009; Zhang et al., 2015). It could not only promote macrophage migration (Wu et al., 2020), but also promote cancer cell invasion, dissemination, and metastasis in various tumors and causes polygonal cells to develop irregular or elongated fibroblastic morphology (Lu et al., 1999; Dai et al., 2011; Huang et al., 2018). In brain tumors, it is associated with the malignant progression of astrocytoma and promotes astrocytoma cell invasion (Wang et al., 2011). Furthermore, delta-catenin might promote bevacizumab-induced glioma invasion (Shimizu et al., 2019).

Prior to now, there has been a poor understanding of the context-dependent roles of delta-catenin in cancer. Two explanations for this have been proposed. First, it may be primarily due to variation in CTNND2. In prostate cancer, wild-type and mutant delta-catenin both exhibited pro-oncogenic and tumor-suppressive functions; that study found a protein truncation, caused by a nonsense mutation of delta-catenin, mainly occurring in the cytoplasm, promoted carcinoma progression *via* various pathways (Li et al., 2020). CTNND2 has a vast number of known mutations; the Sanger COSMIC database has recorded 541 unique mutations of delta-catenin, in 38 different tissues, and some of these mutations alter delta-catenin structure and function in tumors (Lu et al., 2016). This may therefore explain the contrasting reported effects of delta-catenin.

The second possible reason might be related to epigenetic modification. Post-translational phosphorylation of delta-catenin, which alters its function substantially, has been increasingly described. Phosphorylation of delta-catenin plays an important role in determining delta-catenin’s role in neuronal development and oncogenesis (Oh et al., 2009; Poore et al., 2010; Chen et al., 2021). And a classic dual-function model has been identified, involving a phospho-switch in delta-catenin that can trigger two opposite effects on dendrite development (Baumert et al., 2020). Thus, its phosphorylation status may cause these contrasting effects.

Medulloblastoma, which is differ from the other pediatric brain tumor such as ependymoma and brainstem glioma (Duc, 2020; Duc et al., 2020), is the most common malignant brain tumor in children. The challenge in medulloblastoma therapy is to address tumor cell invasion and dissemination, which cannot completely be prevented by treatment currently. Radiotherapy of the total central nervous system and chemotherapy are typically used to avoid tumor cell infiltration and metastasis, but they can cause intellectual and neurological disabilities in survivors (Mabbott et al., 2008). The fact that metastasis is rare in WNT subgroup suggests that unexamined molecular mechanisms in this group may help to prevent medulloblastoma invasion and dissemination. Delta-catenin is enriched in WNT subgroup and associated with lower tumor dissemination in our study. Delta-catenin has been reported promoting β-catenin nuclear localization and activating WNT pathway, and therefore accelerate tumor progression (Nopparat et al., 2015; Huang et al., 2018; Ju et al., 2020). However, WNT activation was found an unexpected tumor suppressor in medulloblastoma in several studies (Pöschl et al., 2014; Zinke et al., 2015; Manoranjan et al., 2020). The role of delta-catenin in WNT activation might be the reason that it is related with better prognosis in medulloblastoma patients according to this unexpected finding.

This study has some certain limitations. The cell line in our study are all belong to SHH subgroup (Ivanov et al., 2016). Given that delta-catenin is enriched in WNT Subgroup, the exploring of delta-catenin function in WNT subgroup cell model would reflect its role in cell invasion precisely. However, there is no WNT cell line available except for primary cells derived from medulloblastoma tissue (Ivanov et al., 2016; Manoranjan et al., 2020), which we would establish and use in further research. Exploring the underlying molecular mechanisms which delta-catenin works in WNT subgroup would also be our future direction. In addition, the validation of delta-catenin’s role in invasion is also lacking in other subgroup cell lines (Group3/4) in our study, which need further validation in the future.

## Conclusion

In conclusion, we found that high expression of delta-catenin, which is enriched in WNT subgroup, is a favorable prognostic factor in medulloblastoma. The EMT pathway may participate in this reduced invasion and metastasis.

## Data Availability

The original contributions presented in the study are included in the article/Supplementary Material, and the raw data of this paper have been uploaded onto the Research Data Deposit (RDD) with an RDD number of RDDB2022275274.
