# Supplementary material for: Delta-catenin attenuates medulloblastoma cell invasion by targeting EMT pathway
Source: Front Genet. 2022 Oct 11;13:867872. doi: 10.3389/fgene.2022.867872 (PMC9595215; doi:10.3389/fgene.2022.867872)
Supplement: Supplementary file 3 [file Table1.docx]

Supplementary Table 1 Details of the GEO datasets included in this study

| **Datasets** | **References** | **Platform** | **Sample size (tumor/control)** | **Application** |
| --- | --- | --- | --- | --- |
| GSE85217 | [Florence MC](https://pubmed.ncbi.nlm.nih.gov/?term=Cavalli+FMG&cauthor_id=28609654)G et al[1]. | [HuGene-1_1-st] Affymetrix Human Gene 1.1 ST Array [transcript (gene) version] | 763 (tumor) | **Explore clinical relevance** |
| GSE30074 | [Park W](https://www.ncbi.nlm.nih.gov/pubmed/?term=Park%20W%5bAuthor%5d) et al[2] . | [HuGene-1_0-st] Affymetrix Human Gene 1.0 ST Array [transcript (gene) version] | 30 (tumor) | **Explore clinical relevance** |
| GSE21104 | Northcott PA et al[3] | [HuEx-1_0-st] Affymetrix Human Exon 1.0 ST Array [transcript (gene) version] | 103 （tumor） | **Explore clinical relevance** |
| GSE73066 | [Zakrzewski K](https://www.ncbi.nlm.nih.gov/pubmed/?term=Zakrzewski%20K%5bAuthor%5d)[4]. | [HG-U133_Plus_2] Affymetrix Human Genome U133 Plus 2.0 Array | 47 (control) | **Validation delta-catenin expression** |
| GSE44971 | [Lambert SR](https://www.ncbi.nlm.nih.gov/pubmed/?term=Lambert%20SR%5bAuthor%5d)[5]. | [HG-U133_Plus_2] Affymetrix Human Genome U133 Plus 2.0 Array | 9 (control) | **Validation delta-catenin expression** |
| GSE10327 | Kool M[6]. | [HG-U133_Plus_2] Affymetrix Human Genome U133 Plus 2.0 Array | 62 (tumor) | **Validation delta-catenin expression** |
| GSE49243 | Pfister S[7]. | [HG-U133_Plus_2] Affymetrix Human Genome U133 Plus 2.0 Array | 73 (tumor) | **Validation delta-catenin expression** |
| GSE50161 | Griesinger AM[8]. | [HG-U133_Plus_2] Affymetrix Human Genome U133 Plus 2.0 Array | 13 (tumor)/5 (control) | **Validation delta-catenin expression** |
| GSE66354 | [Donson AM](https://www.ncbi.nlm.nih.gov/pubmed/?term=Donson%20AM%5bAuthor%5d)[9] | [HG-U133_Plus_2] Affymetrix Human Genome U133 Plus 2.0 Array | 19 (tumor)/2 (control) | **DEGs analysis** |
| GSE86574 | Andrew M Donson[10] | [HG-U133_Plus_2] Affymetrix Human Genome U133 Plus 2.0 Array | 17 (tumor)/5 (control) | **DEGs analysis** |
| GSE74195 | [de Bont JM](https://www.ncbi.nlm.nih.gov/pubmed/?term=de%20Bont%20JM%5bAuthor%5d)[11] | [HG-U133_Plus_2] Affymetrix Human Genome U133 Plus 2.0 Array | 23 (tumor)/5 (control) | **DEGs analysis** |

# REFERENCES

1. Cavalli, F., et al., *Intertumoral Heterogeneity within Medulloblastoma Subgroups.* Cancer cell, 2017. **31**(6): p. 737-754.e6.

2. Park, A., et al., *Prognostic classification of pediatric medulloblastoma based on chromosome 17p loss, expression of MYCC and MYCN, and Wnt pathway activation.* Neuro-oncology, 2012. **14**(2): p. 203-14.

3. Northcott PA., et al. Medulloblastoma comprises four distinct molecular variants. *J Clin Oncol* 2011 Apr 10;29(11):1408-14.

4. Zakrzewski, K., et al., *Transcriptional profiles of pilocytic astrocytoma are related to their three different locations, but not to radiological tumor features.* BMC cancer, 2015. **15**: p. 778.

5. Lambert, S., et al., *Differential expression and methylation of brain developmental genes define location-specific subsets of pilocytic astrocytoma.* Acta neuropathologica, 2013. **126**(2): p. 291-301.

6. Kool, M., et al., *Integrated genomics identifies five medulloblastoma subtypes with distinct genetic profiles, pathway signatures and clinicopathological features.* PloS one, 2008. **3**(8): p. e3088.

7. Pöschl, J., et al., *Genomic and transcriptomic analyses match medulloblastoma mouse models to their human counterparts.* Acta neuropathologica, 2014. **128**(1): p. 123-36.

8. Griesinger, A., et al., *Characterization of distinct immunophenotypes across pediatric brain tumor types.* Journal of immunology (Baltimore, Md. : 1950), 2013. **191**(9): p. 4880-8.

9. Griesinger AM, Josephson RJ, Donson AM, Mulcahy Levy JM et al. Interleukin-6/STAT3 Pathway Signaling Drives an Inflammatory Phenotype in Group A Ependymoma. *Cancer Immunol Res* 2015 Oct;3(10):1165-74.

10. Amani V, Donson AM, Lummus SC, Prince EW et al. Characterization of 2 Novel Ependymoma Cell Lines With Chromosome 1q Gain Derived From Posterior Fossa Tumors of Childhood. *J Neuropathol Exp Neurol* 2017 Jul 1;76(7):595-604.

11. de Bont JM, Kros JM, Passier MM, Reddingius RE et al. Differential expression and prognostic significance of SOX genes in pediatric medulloblastoma and ependymoma identified by microarray analysis. *Neuro Oncol* 2008 Oct;10(5):648-60.
