# Supplementary figures and images for: Delta-catenin attenuates medulloblastoma cell invasion by targeting EMT pathway
Source: Front Genet. 2022 Oct 11;13:867872. doi: 10.3389/fgene.2022.867872 (PMC9595215; doi:10.3389/fgene.2022.867872)

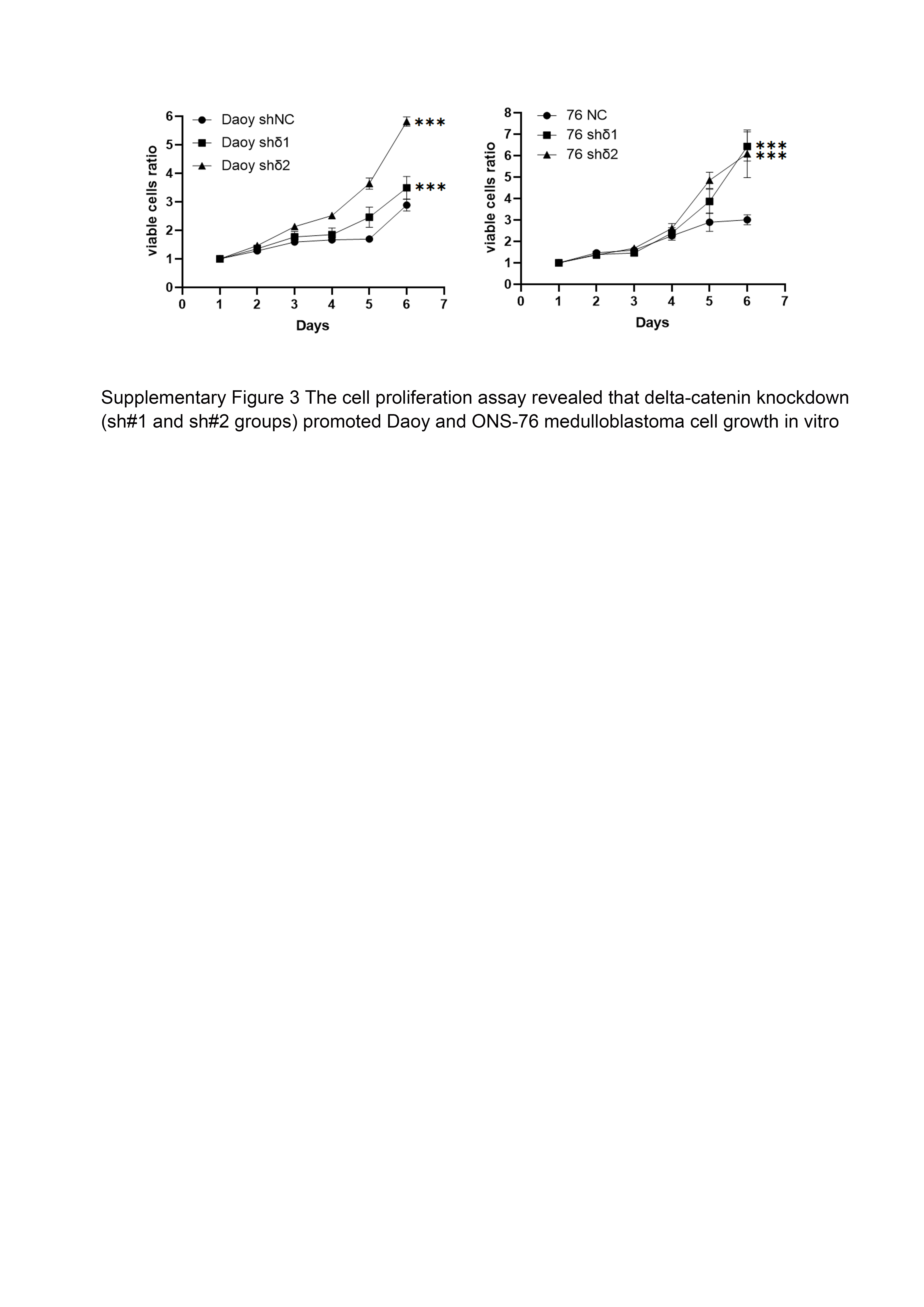

Supplement: Supplementary file 4 [file Image3.TIF]

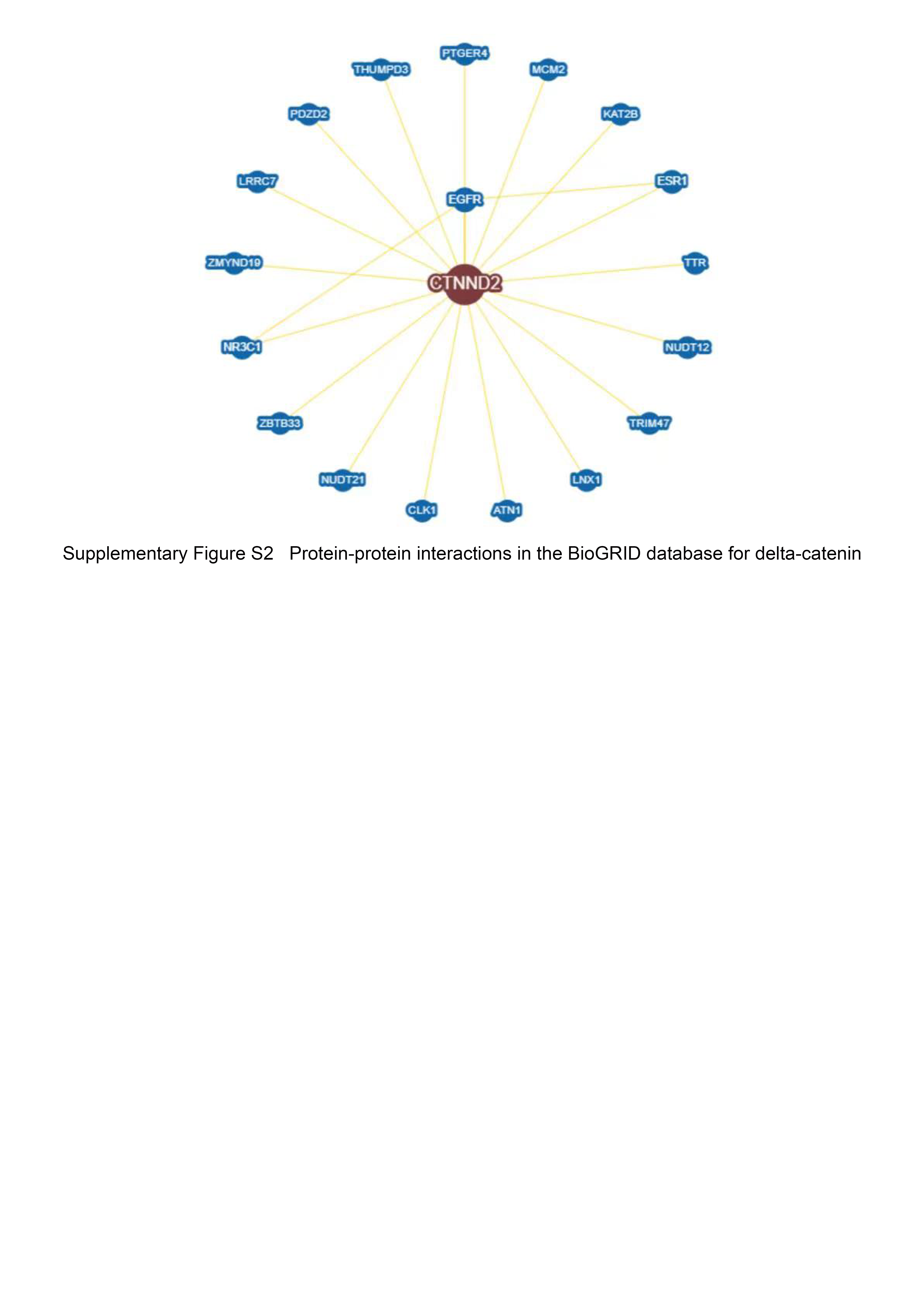

Supplement: Supplementary file 5 [file Image2.TIF]

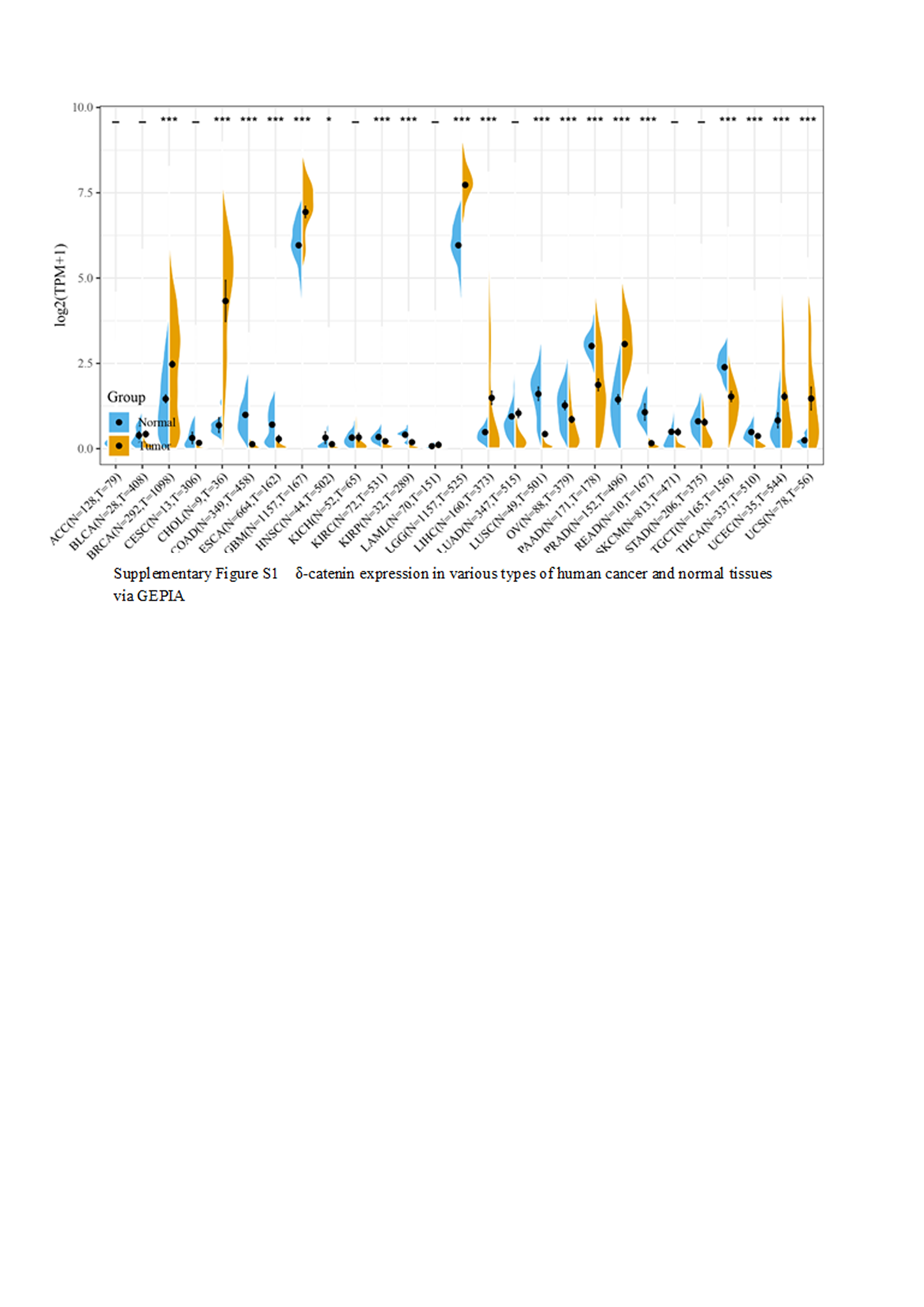

Supplement: Supplementary file 6 [file Image1.TIF]
