# Supplementary material for: Delta-catenin attenuates medulloblastoma cell invasion by targeting EMT pathway
Source: Front Genet. 2022 Oct 11;13:867872. doi: 10.3389/fgene.2022.867872 (PMC9595215; doi:10.3389/fgene.2022.867872)
Supplement: Supplementary file 7 [file DataSheet1.PDF]

中山大学肿瘤防治中心伦理委员会

科研项目审批件

审批号： GZR2017-007　　审批日期：2017 年 2 月 22 日

地址：广州市越秀区东风东路 651 号　邮编：510060　电话：87343135　传真：87343009

基金类型： 国家自然科学基金申请

项目类别： 面上项目

项目名称： WTX 遗传变异在家族性髓母细胞瘤发生发展中的作用研究

申请人： 王　静

依托/承担单位： 中山大学/肿瘤防治中心

审批意见：

本伦理委员会(参加委员名单见附件)于 2017 年 2 月 22 日召开会议，严格按照 ICH-GCP 原则及中国相关的法规/指南，审阅并讨论了该科研项目。应到人数 14 人，其中 4 人因事请假，实到人数 10 人。投票结果：10 位委员均表示“同意”。根据投票结果，本委员会同意自审批之日起同意该项目开展，并要求：所有资料未经本委员会批准，不得作任何修改。

中山大学肿瘤防治中心伦理委员会

主任（签名）：

2017 年 2 月 22 日

项目名称：WTX 遗传变异在家族性髓母细胞瘤发生发展中的作用研究

中山大学肿瘤防治中心伦理委员会成员名单

伦理委员会职务	姓名	性别	职务、职称	专业	签名
主任	彭望清	男	中心纪委书记	行政管理	
副主任	符立梧	男	实验研究部主任<br>研究员	肿瘤药理	/
副主任	王树森	男	内科<br>主任医师	临床医学	
委员	夏云飞	男	放疗科<br>教授	临床医学	/
委员	钟志勇	男	天河区五山街道电子五所社区居民委员会委员	行政管理	
委员	陈　功	男	结直肠科<br>副主任医师	临床医学	/
委员	曹蔚玮	女	中心办副主任<br>助理研究员	行政管理	
委员	吴海鹰	男	内科<br>副主任医师	临床医学	/
委员	何　韵	男	医务处处长	行政管理	
委员	张 阳	男	临床研究部<br>I 期病房<br>主治医师	临床医学	
委员	覃惠英	女	护理部主任<br>主任护师	护理学	
委员	徐　立	女	肝胆科<br>主任医师	临床医学	
委员	周昕熙	女	科教处<br>副研究员	肿瘤学	
委员	刘孟斌	男	广东三环汇华律师事务所所长、律师、副研究员	法律	

日期：2017年2月22日

中山大学肿瘤防治中心伦理委员会
